# Contribution of transient and sustained calcium influx, and sensitization to depolarization-induced contractions of the intact mouse aorta

**DOI:** 10.1186/1472-6793-12-9

**Published:** 2012-09-03

**Authors:** Paul Fransen, Cor E Van Hove, Johanna van Langen, Dorien M Schrijvers, Wim Martinet, Guido R Y De Meyer, Hidde Bult

**Affiliations:** 1 Laboratory of Physiopharmacology, University of Antwerp, Universiteitsplein 1 Building T, 2.18, Wilrijk B-2610, Belgium; 2 Laboratories of Pharmacology, University of Antwerp, Antwerp, Belgium

**Keywords:** Vascular smooth muscle, L-type Ca^2+^ channel, Vasoconstriction, Intracellular Ca^2+^, Depolarization, Window Ca^2+^ influx

## Abstract

**Background:**

Electrophysiological studies of L-type Ca^2+^ channels in isolated vascular smooth muscle cells revealed that depolarization of these cells evoked a transient and a time-independent Ca^2+^ current. The sustained, non-inactivating current occurred at voltages where voltage-dependent activation and inactivation overlapped (voltage window) and its contribution to basal tone or active tension in larger multicellular blood vessel preparations is unknown at present. This study investigated whether window Ca^2+^ influx affects isometric contraction of multicellular C57Bl6 mouse aortic segments.

**Results:**

Intracellular Ca^2+^ (Ca_i_^2+^, Fura-2), membrane potential and isometric force were measured in aortic segments, which were clamped at fixed membrane potentials by increasing extracellular K^+^ concentrations. K^+^ above 20 mM evoked biphasic contractions, which were not affected by inhibition of IP_3_- or Ca^2+^ induced Ca^2+^ release with 2-aminoethoxydiphenyl borate or ryanodine, respectively, ruling out the contribution of intracellular Ca^2+^ release. The fast force component paralleled Ca_i_^2+^ increase, but the slow contraction coincided with Ca_i_^2+^ decrease. In the absence of extracellular Ca^2+^, basal tension and Ca_i_^2+^ declined, and depolarization failed to evoke Ca_i_^2+^ signals or contraction. Subsequent re-introduction of external Ca^2+^ elicited only slow contractions, which were now matched by Ca_i_^2+^ increase. After Ca_i_^2+^ attained steady-state, isometric force kept increasing due to Ca^2+^- sensitization of the contractile elements. The slow force responses displayed a bell-shaped voltage-dependence, were suppressed by hyperpolarization with levcromakalim, and enhanced by an agonist of L-type Ca^2+^ channels (BAY K8644).

**Conclusion:**

The isometric response of mouse aortic segments to depolarization consists of a fast, transient contraction paralleled by a transient Ca^2+^ influx via Ca^2+^ channels which completely inactivate. Ca^2+^ channels, which did not completely inactivate during the depolarization, initiated a second, sustained phase of contraction, which was matched by a sustained non-inactivating window Ca^2+^ influx. Together with sensitization, this window L-type Ca^2+^ influx is a major determinant of basal and active tension of mouse aortic smooth muscle.

## Background

Transcripts and protein expression of the Ca^2+^ channel gene are found widely in the cardiovascular system, where the channels play a dominant role in blood pressure regulation [1-5]. This regulation not only occurs via modulation of peripheral resistance, but also via determination of the arterial compliance, especially in old age (systolic) hypertension [6-8]. It has been shown that L-type Ca^2+^ channel blockers increase vascular compliance of large elastic vessels. As such, they may also be of importance for the pathogenesis and prognosis of cardiovascular complications such as atherosclerosis, left ventricular hypertrophy and heart failure [8-14]. Vascular reactivity via L-type Ca^2+^ influx is often studied by increasing the extracellular K^+^ and depolarizing the cells membrane potential (V_m_). High K^+^ induces biphasic contractions in rabbit arteries [15], rat basilar arterial rings [16] and mouse aorta [17], whereby the tonic rise in force is actually accompanied by a decline of intracellular Ca^2+^. This is often attributed to Ca^2+^-sensitization, whereby suppression of myosin light chain phosphatase activity raises contractile force independently of further increases or even decrease in intracellular Ca^2+^[15,18-21]. In those studies, however, relationships between force and continuous background Ca^2^ influx via non-inactivating L-type Ca^2+^ channels were not explored.

Indeed, (electro)physiological characteristics of L-type Ca^2+^ channels, which have been studied extensively in isolated cardiomyocytes and vascular smooth muscle cells (VSMCs), are such that voltage-dependent activation and inactivation curves show substantial overlap between −40 and −15 mV revealing a time-independent, but voltage-dependent Ca^2+^ influx (window current) in isolated cells [22-26]. Although pharmacological evidence suggested that this window may at least serve as a background Ca^2+^ influx pathway responsible for myogenic tone of small arteries, coronary arteries and microvascular resistance vessels [27-29], window Ca^2+^ currents and related window intracellular Ca^2+^ signals have only been determined in voltage-clamped isolated SMCs and not in multicellular vascular tissue [24]. The present study used aortic segments of C57Bl6 mice to investigate relationships between VSMC Ca^2+^ mobilization and isometric contraction with focus on the L-type Ca^2+^ channel window. Since electrophysiological voltage-clamp of intact aorta segments was impossible, we decided to clamp the membrane potential at fixed potentials by increasing external K^+^ concentration. By modulating influx of Ca^2+^ before and during depolarization, we show that not only basal tension, but also the tonic contractile component of C57Bl6 mouse aortic VSMCs depends on the window L-type Ca^2+^ influx and subsequent Ca^2+^ sensitization mechanisms. These observations may have important consequences for the effects of nitric oxide (NO) on L-type Ca^2+^ influx. Recently, we showed that the relaxing efficacy of NO in mouse aorta was dependent on the contractile agonist, and more specifically, decreased when the contraction was mainly elicited via L-type Ca^2+^ influx as with elevated extracellular K^+^, but increased when Ca^2+^ influx was partially inhibited with L-type Ca^2+^ channel blockers [30].

## Results

### Contraction at depolarized potentials

**Figure 1:**
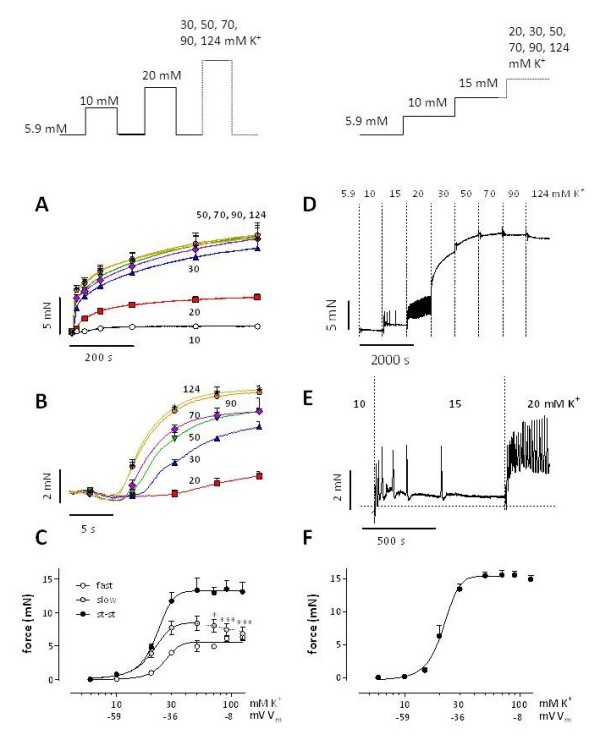
**Isometric contractions by elevation of external K**^**+**^**in mouse aorta.** K^+^ was elevated from 5.9 mM to 10, 20, 30, 50, 70, 90 or 124 mM K^+^ according to the protocols shown in the top panels. For the repetitive protocol (**A-C**), traces, shown on condensed (**A**) and expanded (**B**) time scales, were analyzed with a bi-exponential function revealing [K^+^]-force curves for the fast, slow and steady-state (st-st) force components (**C**). For the cumulative protocol (**D-F**), **D** shows a representative example of isometric force elicited by gradual elevations of extracellular K^+^. **E** displays the 5.9 to 10, 10 to 15 and 15 to 20 mM K^+^ depolarizations on an expanded time scale. In **F** “steady-state” force at each step was plotted in function of [K^+^]. Results show mean ± s.e.m, n = 4 (**A-C)** or n = 5 (**F**). *, ***: P<0.05, 0.001 decrease of slow component amplitude versus maximum at 50 mM K^+^. The estimated values of V_m_ at 10, 30 and 100 mM [K^+^] are indicated. These values are respectively −59, -36 and −8 mV (see Additional file 1).

Membrane potentials (V_m_) in intact mouse aortic VSMCs were K^+^-dependent and depolarised from −60 mV at 5.9 mM K^+^ to −30 mV at 50 mM K^+^ (see Additional file 1). Hence, elevation of extracellular K^+^ is a good method to clamp multicellular aortic segments from resting potentials at 5.9 mM K^+^ to depolarized potentials. Two K^+^ clamp protocols, as shown in Figure 1, were used; they differed in the relative number of L-type Ca^2+^ channels that can be activated with the subsequent depolarization. In the repetitive protocol (Figure 1 A-C), which mimics the depolarizing voltage steps in voltage-clamp experiments of single VSMCs, segments at 5.9 mM K^+^ were repetitively exposed to elevated K^+^ followed by return to 5.9 mM K^+^. In this protocol, the number of channels that can be activated by the depolarization step is always the same at the start of the depolarization. In the cumulative protocol (Figure 1 D-F), which mimics the variable holding potentials in voltage-clamp experiments in single VSMCs, the segments were depolarized to the subsequent higher K^+^ concentration without return to 5.9 mM K^+^. Therefore, with this protocol the relative number of Ca^2+^ channels that can be activated with the subsequent depolarization decreases with higher K^+^.

Isometric force by the repetitive protocol followed a bi-exponential time course, except at 10 mM K^+^ (Figure 1A). Amplitude (Figure 1B) and velocity of the fast component increased with the K^+^ concentration (time constant 27.1 ± 6.0 s at 20 mM K^+^, 3.8 ± 0.7 s at 124 mM K^+^, P<0.001). The amplitude of the slow component showed a maximum around 50 mM K^+^, but then significantly decreased at 90 and 124 mM K^+^ (Figure 1C). Remarkably, its time constant was independent of external K^+^ (258 ± 34 s at 20 mM K^+^ and 253 ± 27 s at 124 mM K^+^). [K^+^]-force relationships (Figure 1C) revealed E_max_-values of 6.1 ± 0.5, 9.4 ± 1.3 and 14.4 ± 1.6 mN for fast, slow and steady-state force. EC_50_ values were respectively 23.5 ± 1.2, 22.2 ± 0.3 and 22.0 ± 0.3 mM K^+^ and were not significantly different.

In the cumulative protocol, two force signals were seen at 15 and 20 mM K^+^: on top of a tonic rise upon depolarization, transient force spikes were observed (Figure 1D and E). These spikes faded away as time progressed (15 mM K^+^), and showed increased frequency, but similar amplitudes at 20 mM K^+^. At 30 and 50 mM K^+^ these spikes disappeared, but force developed with a fast and slow component. Above 50 mM K^+^ only a small increase (50–70 mM K^+^) or even a decrease (90–124 mM K^+^) of force was observed (Figure 1D). E_max_ (15.5 ± 0.6 mN, Figure 1F) and EC_50_ (21.8 ± 1.2 mM K^+^) were not significantly different from the steady state values measured with repetitive depolarization (*vide supra*).

**Figure 2:**
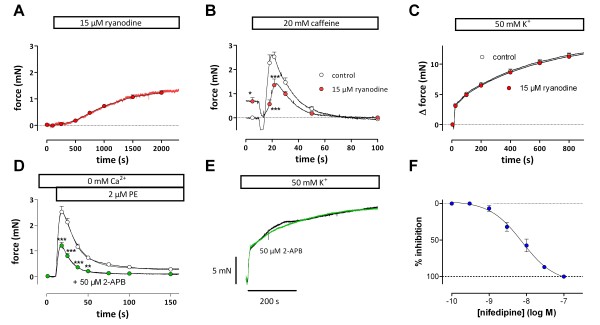
**Ca**^**2+ **^**from intracellular Ca**^**2+**^**stores does not contribute to K**^**+**^**contractions.** Effects of 15 μM ryanodine on baseline isometric tension (**A**), and on force evoked by 20 mM caffeine (**B**) or 50 mM K^+^ (**C**). Results show mean ± s.e.m, n = 4. *, ***: P<0.05, 0.001, control versus ryanodine. Effects of 50 μM 2-APB on contraction induced by 2 μM PE in 0Ca solution (**D**) or contraction induced by 50 mM K^+^ (**E**). Results show mean ± s.e.m, n = 4. **, ***: P<0.01, 0.001, control versus 2-APB. (**F**) Dose–response curve for inhibition by nifedipine of the contractions evoked by 50 mM K^+^ (n = 4).

Neurotransmitter release from perivascular nerves did not contribute to the biexponential nature of high K^+^ contractions or to the K^+^-dose–response relationships in aortic segments (see Additional file 1). There was also no evidence of involvement of sarcoplasmic reticulum (SR) Ca^2+^ store Ca^2+^ release. Although inhibition of Ca^2+^-induced Ca^2+^ release with 15 μM ryanodine raised basal tension (Figure 2A), and inhibited the transient caffeine-induced contraction by more than 50% (Figure 2B), 50 mM K^+^-induced contractions were not affected (Figure 2C). Similar observations were made for inositoltriphosphate (IP_3_)-mediated Ca^2+^ release. Contractions by 2 μM phenylephrine (PE) in the absence of extracellular Ca^2+^ were significantly reduced by 50 μM 2-aminoethoxydiphenyl borate (2-APB), a blocker of IP_3_-induced Ca^2+^ release [31] (Figure 2D), whereas contractions by 50 mM K^+^ were not affected (Figure 2E). 

Moreover, K^+^ in Ca^2+^-free KR (0Ca) or in the presence of 3 μM nifedipine, an inhibitor of L-type Ca^2+^ channels, failed to elicit tension, and addition of nifedipine (3 to 300 nM) to segments constricted with 50 mM K^+^ caused complete relaxation (E_max_ 107 ± 3%, logEC_50_ -8.12 ± 0.12, n = 4, Figure 2F). Finally, inhibition of SERCA and emptying the intracellular Ca^2+^ stores with 1 μM cyclopiazonic acid (CPA) did not affect the contraction by 50 mM K^+^.

These results indicated that SR Ca^2+^ is not involved in K^+^-evoked contractions and that fast and slow force components evoked by high K^+^ were both initiated and sustained by Ca^2+^ influx via VSMC L-type Ca^2+^ channels only.

### Relationship between force and Ca^2+^ influx

**Figure 3:**
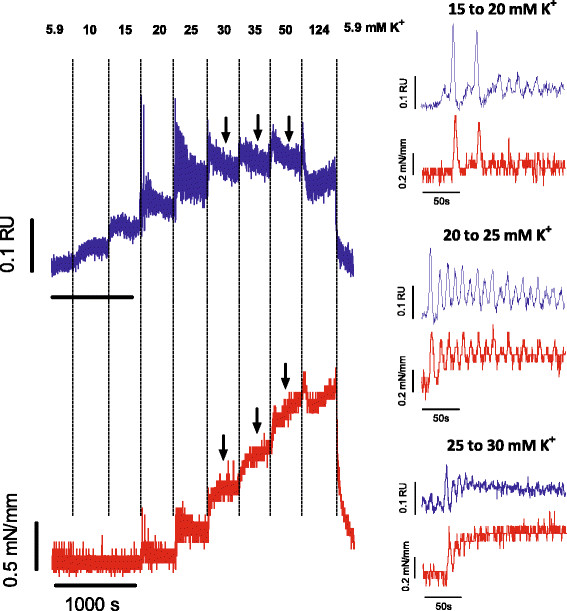
**Depolarisation with elevated K**^**+ **^**induces force and intracellular Ca**^**2+**^** signals.** Representative example (n = 3) of intracellular Ca^2+^ (ratio 340/380, RU, blue) and isometric force (red) of an aortic segment upon gradual elevation of external K^+^ from 5.9 mM to the values indicated on top of the figure. Both signals are shown in the right panel on an extended time scale for transitions from 15 to 20, from 20 to 25 and from 25 to 30. Black arrows indicate decrease of Ca_i_^2+^ with accompanying increase of force.

Temporal relationships between intracellular Ca^2+^ and isometric force were explored using the cumulative protocol. For K^+^ elevations from 15 to 20, from 20 to 25 and from 25 to 30 mM K^+^, there was a strict temporal relationship between Ca^2+^ and force (Figure 3). Again, there were tonic and phasic contractions (cf Figure 1D and E), though at slightly higher K^+^ concentrations (20–30 mM K^+^). They coincided with phasic Ca^2+^ spikes on top of a tonic rise of Ca^2+^ (Figure 3). Both Ca^2+^ and force spikes faded away as time progressed (15 to 20 mM K^+^), displayed higher frequency at the subsequent step (25 mM K^+^) and disappeared at holding potentials above 30 mM K^+^. From 35 up to 124 mM K^+^ the temporal relationships between Ca^2+^ (transient peak tapering off to lower plateau) and force (biphasic increase) were not clear and during these depolarizations Ca^2+^ decreased whereas force increased (arrows in Figure 3).

**Figure 4:**
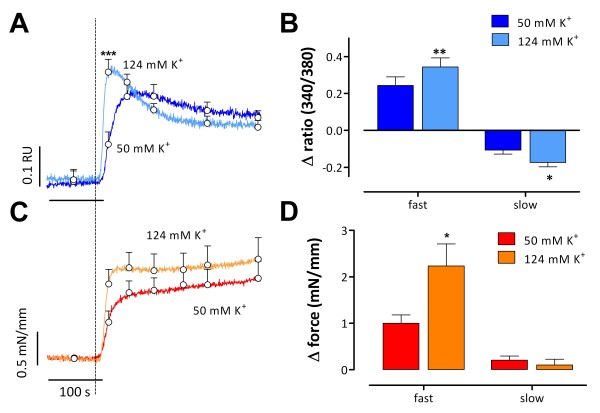
**Temporal relationships between force and intracellular Ca**^**2+**^** signals at elevated K**^**+**^**.** Intracellular Ca^2+^ (ratio 340/380, RU, blue, **A**) and force (red, **C**) signals upon depolarization from 5.9 mM K^+^ to 50 or 124 mM K^+^. Ca^2+^ and force signals consisted of fast and slow components with amplitudes shown in **B** and **D**. Results show mean ± s.e.m, n = 6. *, **, ***: P<0.05, 0.01, 0.001 for 124 versus 50 mM K^+^.

The deviations between Ca^2+^ and force above 30 mM K^+^ were studied in greater detail by depolarizing the segments from 5.9 mM K^+^ to 50 or 124 mM K^+^. The initial, fast contraction was accompanied by a fast rise in Ca^2+^ (Figure 4). Amplitude and velocity of the fast Ca^2+^ (7.2 ± 1.5 s) and force (7.7 ±1.2 s) components were greater at 124 mM K^+^ as compared with 50 mM K^+^ (16.8 ± 3.7 s and 14 ± 3 s respectively). After reaching a maximum, Ca^2+^ declined faster (50 ± 6 s versus 137 ± 24 s, P<0.01) and to a lower level at 124 mM K^+^ than at 50 mM K^+^, and the slow force increase during the plateau phase was slightly smaller at 124 mM K^+^.

These results indicate that at 50 or 124 mM K^+^ the slow contraction was actually accompanied by a decline of Ca^2+^, but that there was a good temporal relationship between intracellular Ca^2+^ and force development immediately after the depolarization.

### Experimental dissection of the Ca^2+^ and force components

**Figure 5:**
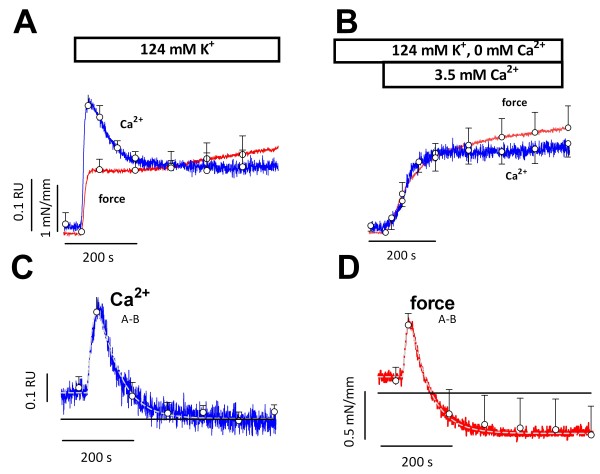
**Depolarization in the absence of external Ca**^**2+**^** eliminates the fast, transient Ca**_**i**_^**2+**^** and force signals.** Intracellular Ca^2+^ (ratio 340/380, blue) and force (red) upon depolarization from 5.9 mM K^+^ to 124 mM K^+^ in the presence of 2.5 mM external Ca^2+^ (124 mM K^+^, **A**) or after re-addition of 3.5 mM Ca^2+^ to 0Ca (124 K^+^/0-3.5Ca, **B**). Pair-wise subtraction of 124 mM K^+^/0-3.5Ca from 124 mM K^+^ signals yielded the differential curves for Ca^2+^ (**C**) and force (**D**). Results show mean ± s.e.m, n = 4.

At 5.9 mM K^+^, removal of extracellular Ca^2+^ (0Ca) decreased basal intracellular Ca^2+^ and force from 0.91 ± 0.03 to 0.81 ± 0.02 RU (p<0.005, n = 6) and from 0.52 ± 0.02 to 0.40 ± 0.05 mN/mm (p<0.05, n = 6), indicating baseline Ca^2+^ influx via Ca^2+^ channels in normal conditions. Depolarizing the segments with 124 mM K^+^ in 0Ca abrogated Ca^2+^ influx via L-type Ca^2+^ channels and neither contraction nor Ca^2+^ influx was observed. Because in the absence of extracellular Ca^2+^ L-type Ca^2+^ channels display normal gating currents [32], subsequent addition of external Ca^2+^ can evoke Ca^2+^ influx and contraction only if a subpopulation of L-type Ca^2+^ channels is not completely inactivated during the preceding depolarization in 0Ca. Indeed, re-addition of Ca^2+^ to 0Ca caused intracellular Ca^2+^ and force to increase (Figure 5B). Contrary to the control situation (Figure 5A), intracellular Ca^2+^ did not decline during the contraction plateau in the Ca^2+^ re-addition experiments. As a consequence, a clear temporal relationship between the slow Ca^2+^ and force signals was observed (Figure 5B) and the force and Ca^2+^ signals could now be dissected in parallel fast and slow components. 

The fast Ca^2+^ and force components that were eliminated in the Ca^2+^ re-addition experiments, could be visualized by pair-wise subtracting Ca^2+^ and force traces from control traces (Figure 5C, D). The differential Ca^2+^ and force signals displayed a similar time-dependency (time constants respectively 15 ± 2 s and 9 ± 2 s for rise, and 57 ± 4 s and 54 ± 4 s for fall). Therefore, Figures 4 and 5 illustrate the strict temporal relationships between fast and slow Ca^2+^ and force signals upon depolarization: the fast transient Ca^2+^ increase during depolarization initiates fast force development, whereas a simultaneously activated slower influx of Ca^2+^ is responsible for sustained force development during the plateau phase.

### Is L-;type Ca^2+^ window current responsible for the slow contraction phase?

**Figure 6:**
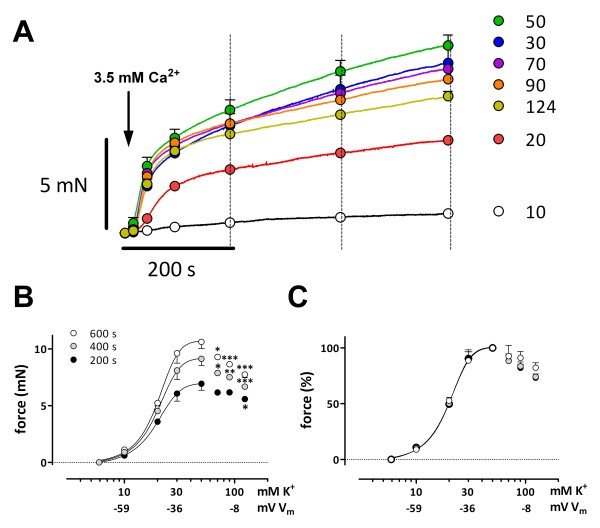
**K**^**+**^**-dependent development of window contraction.** Isometric contractions induced by addition of 3.5 mM Ca^2+^ to 0Ca containing 10 up to 124 mM K^+^ (**A**). Absolute (**B**) and relative (50 mM K^+^ set to 100%, **C**) [K^+^]-force curves were determined at 200, 400 and 600 s (see dotted lines in **A**), were bell-shaped and could only be fitted up to 50 mM K^+^ (**B, C**). Results show mean ± s.e.m, n = 6. *, **, ***: P<0.05, 0.01, 0.001 versus 50 mM K^+^.

An important electrophysiological property of L-type Ca^2+^ channels is that in the voltage range where activation and inactivation curves overlap, they allow a continuous, time-independent Ca^2+^ influx, the so-called window L-type Ca^2+^ channel current [24,26]. If this current is responsible for the slow contraction phase following addition of external Ca^2+^ to segments depolarized in 0Ca, then force should display a bell-shaped concentration-response relationship. Figure 6 shows the contractions evoked by re-introduction of Ca^2+^ to 0Ca at different K^+^ concentrations. After 200 s the slow component showed a linear rather than exponential increase with time. Force measured at 600 s was maximal at 50 mM K^+^ and declined at higher K^+^ concentrations (Figure 6A). 

**Figure 7:**
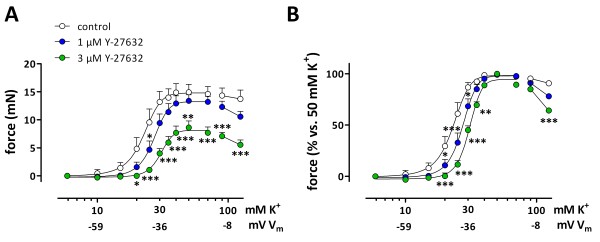
**Effects of Rho kinase inhibition with Y-27632 on window contractions. A**: “Steady-state” isometric contractions induced by depolarizations with cumulative K^+^ concentrations in the absence (control) and in the presence of 1 and 3 μM Y-27632. In **B**, force was normalized with values at 50 mM K^+^ as 100%. Results show mean ± s.e.m, n = 4. *, **, ***: P<0.05, 0.01, 0.001 versus control.

[K^+^]-contraction curves were determined after 200, 400 and 600 s (Figure 6B and C). At these time intervals, the [K^+^]-contraction curve indeed became bell-shaped. The bell-shape and the complete inhibition with the L-type Ca^2+^ channel blocker, nifedipine (data not shown) are typical characteristics of the window L-type Ca^2+^ current. The EC_50_ for K^+^ was time-independent and was respectively 20.9 ± 0.4 mM, 20.4 ± 0.2 mM and 20.5 ± 0.2 mM (n = 6). The continuous increase of force with time is presumably due to Ca^2+^ sensitization as intracellular Ca^2+^ reached steady-state after 200 s (Figure 5). Further evidence for Ca^2+^ sensitization was provided by Rho kinase inhibition with Y-27632 (1 and 3 μM). Y-27632 attenuated depolarization-induced contractions, but inhibition of Ca^2+^ sensitization emphasized the bell shape of the [K^+^]-contraction curve even more. This suggests that the decrease of force at 90 and 124 mM K^+^ was not due to a reduction in sensitivity to Ca^2+^, but was proportional to the window influx of Ca^2+^ via L-type Ca^2+^ channels (Figure 7). Similar results were obtained with HA 1077 (5 μM, not shown).

### Modulation of L-type window Ca^2+^ influx

**Figure 8:**
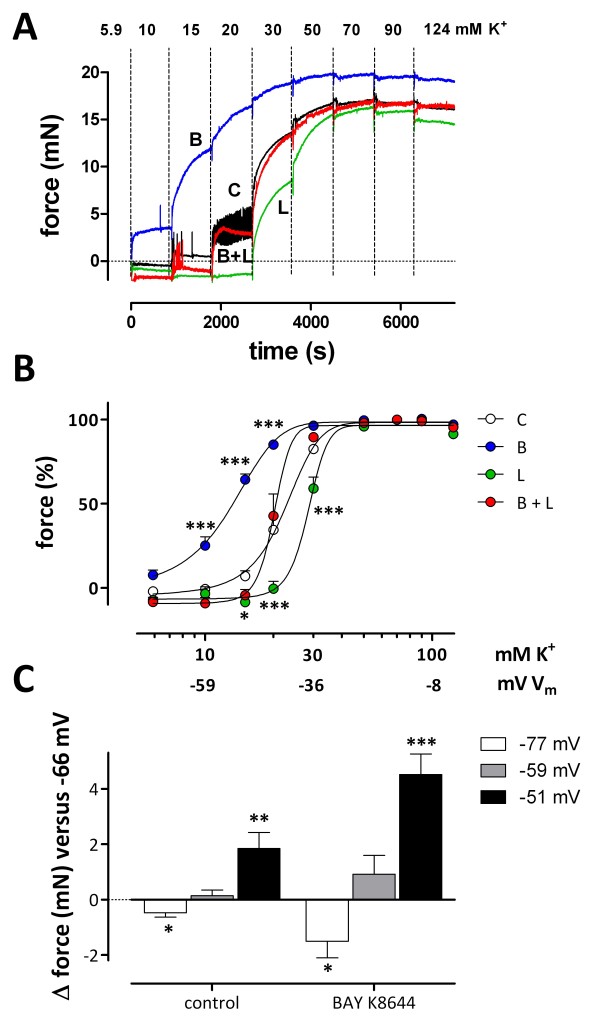
**Stimulation and inhibition of window contraction. A**: Representative example of isometric contractions of a segment depolarized with cumulative K^+^ concentrations in the absence (C, black) and presence of 30 nM BAY K8644 (B, blue), 200 nM levcromakalim (L, green), or their combination (B + L, red). **B**: “Steady-state” force at each step was plotted as function of [K^+^] with values at 50 mM K^+^ as 100%. Results show mean ± s.e.m, n = 4. *, ***: P<0.05, 0.001 versus control. **C**: Change of basal tension (Δ force in mN) for repolarization and depolarization of V_m_ by changing extracellular K^+^ to attain V_m_ within the physiological range for non-stimulated VSMCs (K^+^ from 5.9 mM to 2 (white) or 10 (grey) or 15 (black) mM) in control conditions (C) and in the presence of 30 nM BAY K8644 (B). Instead of the actual K^+^ concentration, the estimated V_m_ of the VSMCs is indicated: -77 mV for 2 mM, -66 mV for 5.9 mM, (not shown), -59 mV for 10 mM and −51 mV for 15 mM K^+^. Results show mean ± s.e.m, n = 5. *, **, ***: P<0.05, 0.01, 0.001 versus 5.9 mM K^+^.

Changes of V_m_ of the VSMCs or changes of the voltage-dependent parameters of L-type Ca^2+^ channel gating (activation or inactivation) are expected to affect Ca^2+^ influx and contraction of the segments. Segments could be hyperpolarized from −60 mV to the K^+^ equilibrium potential (V_K_) of −86 mV at 5.9 mM K^+^ with levcromakalim (200 nM), an opener of ATP-dependent K^+^ channels (see Additional file 1, Figure 1). The L-type Ca^2+^ channel activation curve can be shifted to hyperpolarized potentials with BAY K8644 (30 nM), an activator of L-type Ca^2+^ channels [30,33,34]. When segments were subjected to increasing K^+^ concentrations in the presence of levcromakalim, BAY K8644, or their combination (Figure 8A and B), levcromakalim shifted the curve to higher K^+^ concentrations (+5.93 ± 0.87 mM), whereas BAY K8644 caused a shift to lower K^+^ concentrations (-7.98 ± 1.09 mM). Both effects were fully additive, indicating independent effects of V_m_ (levcromakalim) and L-type Ca^2+^ channel gating (BAY K8644) on window contractions. 

At normal extracellular K^+^, levcromakalim caused a glibenclamide (inhibitor of ATP-sensitive K^+^ channels)-sensitive decline of intracellular Ca^2+^ (−0.042 ± 0.012 RU, n = 3) and baseline tension (−0.56 ± 0.28 mN, n = 4), whereas BAY K8644 raised resting intracellular Ca^2+^ (+0.016 ± 0.008 RU) and force (+1.77 ± 0.51 mN, n = 4). The BAY effect could be reversed by addition of levcromakalim or nifedipine (data not shown). To illustrate the physiological importance of the window Ca^2+^ influx for basal contraction of mouse aortic segments, the external K^+^ concentration was changed to obtain depolarizations or repolarizations within the physiological range of V_m_ for VSMCs (Figure 8C). Changes of the extracellular K^+^ between 2 and 15 mM and V_m_ between −77 and −51 mV caused significant alterations of basal force in control, which could be amplified by adding 30 nM BAY K8644 or removed by adding 200 nM levcromakalim (data not shown). These data provide further evidence for the importance of window Ca^2+^ influx within the physiological range of V_m_ or K^+^ concentrations [35,36].

## Discussion and conclusions

The present study showed that the main determinant of depolarization-induced contractions of the mouse aorta was the influx of extracellular Ca^2+^ via L-type Ca^2+^ channels. Thereby, both Ca^2+^ influx and contraction depended on the amplitude of depolarization (reflected by the increase of external K^+^) and on the resting potential of the VSMC (concentration of external K^+^ at the start of depolarization). At resting membrane potentials, elevation of extracellular K^+^ above 10–20 mM caused biphasic contractions and Ca^2+^ signals. Although the relationships between intracellular Ca^2+^ and force appeared to be complex and sometimes non-linear (slow component), we demonstrated that the fast, phasic force component was related to a transient Ca^2+^ influx, presumably via a population of L-type Ca^2+^ channels which activated and completely inactivated during the depolarization. On the other hand, the slow, tonic force component displayed a bell-shaped voltage (K^+^)-dependence and could be attributed to voltage-dependent, “steady state” Ca^2+^ influx via a population of L-type Ca^2+^ channels. These channels did not completely inactivate during sustained depolarization and gave rise to a window contraction. In addition to the Ca^2+^ influx via both populations of L-type Ca^2+^ channels, a time-dependent Ca^2+^ sensitization contributed to the depolarization-induced contractions of the mouse aorta.

### Depolarization-induced contraction is due to activation of L-type Ca^2+^ channels and not to Ca^2+^ release from the SR

As expected [37], contractions induced by high K^+^ were mainly due to influx of extracellular Ca^2+^ via L-type Ca^2+^ channels in the mouse aorta. Firstly, depolarization in the absence of external Ca^2+^ did not elicit intracellular Ca^2+^ signals or contractions. Secondly, selective L-type Ca^2+^ channel blockade (3 μM nifedipine) completely inhibited K^+^-induced contractions. Thirdly, BAY K8644, an agonist of L-type Ca^2+^ channels, increased the K^+^-sensitivity of the contractions. Finally, although intracellular Ca^2+^ release or Ca^2+^-induced Ca^2+^ release through activation of IP_3_ or ryanodine receptors or Ca^2+^ re-uptake to the SR have been shown to contribute to K^+^-induced contractions [16,37,38], this was not observed in mouse aorta segments (see also [24]). Hence, intracellular Ca^2+^ release did not account for the biphasic pattern of high K^+^-induced force and Ca_i_^2+^ and either phase was solely initiated by L-type Ca^2+^ influx.

### Relationships between fast and slow contraction phases and Ca^2+^ influx

The contraction elicited by depolarization of VSMCs has been studied extensively [39], but has never been directly correlated with the known electrophysiological properties of L-type Ca^2+^ channels. L-type Ca^2+^ currents in isolated SMCs of various tissues and species display a bell-shaped voltage-dependence with maximal currents at 0 to +20 mV [1,2,14,22,23,25,40]. Activation (opening) of L-type Ca^2+^ channels starts at −50 to −40 mV with half-maximal activation at −30 mV [25,41], whereas inactivation starts at −60 mV, is half maximal at about −30 mV and complete at 0 mV [23,25,41,42]. As a consequence, at voltages between current activation (around −45 mV, 20 to 25 mM K^+^) and complete current inactivation (around 0 mV, 124 mM K^+^), two populations of L-type Ca^2+^ channels are expected to contribute to Ca^2+^ influx and contraction. One population of channels will activate and completely inactivate during the depolarization leading to a transient Ca^2+^ influx and concomitant contraction (see Figure 5C and D). This contraction corresponds with the fast phase of contraction as described in Figure 1C, where it was elicited by step depolarizations of V_m_ by sudden increase of K^+^ from 5.9 mM to values above 20 mM. The physiological importance of these events in VSMCs can be questioned. However, in some experiments (Figure 3), fast time- and voltage-dependent intracellular Ca^2+^ and force spikes appeared on top of a slow rise in tone or Ca^2+^ at 15 to 20 mM K^+^ (−50 to −44 mV), which is near the activation voltage of L-type Ca^2+^ channels and within the physiological range of VSMCs V_m_. As their spiking frequency increased with the amplitude of the depolarization step, fusing to a single fast component at 30 and 50 mM K^+^ (−36 and −24 mV) similar to the fast component in the step protocol, these events might be related with activation and complete inactivation of L-type Ca^2+^ channels. Because they occur at physiological V_m_ of VSMCs, they may have physiological importance. They may be related with the persistent calcium sparklets that are increased in hypertension [43], with artery vasospasm [44] or other pathophysiological processes.

However, at all K^+^ concentrations studied, a variable population of channels will not completely inactivate and will permit “time-independent” Ca^2+^ influx via the so-called voltage window [24]. Hence, every depolarization positive to −45 mV (± 20 mM K^+^) should activate a time-independent, non-inactivating Ca^2+^ influx. Following removal of the fast force component by depolarization in the absence of external Ca^2+^ and, then, re-adding Ca^2+^ (Figures 5 and 6) we demonstrated that this “window” contraction showed a close temporal relationship with the increase of intracellular Ca^2+^ via window L-type Ca^2+^ influx. The electrophysiological characteristics of the L-type Ca^2+^ channel window, i.e. maximal Ca^2+^ influx at −30 mV (40 to 50 mM K^+^) and a bell-shaped voltage-dependence are paralleled by a tonic force component which increased with [K^+^ up to 50 or 70 mM (V_m_ = −20 to −30 mV), but decreased again above 70 mM K^+^, leading to a bell-shaped [K^+^-contraction curve. Its voltage range is bounded at negative potentials by channel activation and at more positive potentials by channel inactivation. This agrees with the K ^+^ -dependence of the slow force component described in Figure 1C and Figure 6.

### Manipulation of the window Ca^2+^ influx and contraction

Our experiments predict that basal force by aortic segments will depend on V_m_ and that changes of V_m_ within the voltage range of the L-type Ca^2+^ channel window will stimulate or inhibit Ca^2+^ influx via L-type Ca^2+^ channels and the concomitant contraction. Since removal of extracellular Ca^2+^ led to a decline of intracellular Ca^2+^ and basal tension in the VSMC of the mouse aorta, a “window” Ca^2+^ influx appeared to be operative and functional at resting potentials, which are between −40 to −60 mV [45,46]. As a consequence, a small decrease (2 mM K^+^, repolarization) or increase (10 mM K^+^, depolarization) of external K^+^ modulates basal tension of the mouse aortic segments, probably via closing and opening of L-type Ca^2+^ channels because the effects of K^+^ changes are emphasized by applying BAY K8644 (Figure 8C).

Hyperpolarization of V_m_, as with EDHF [35,36,47-49] or with K_ATP_ channel openers such as levcromakalim (present study) or cromakalim [45], or with reduction of extracellular K^+^ might pull V_m_ out of the window, thereby decreasing L-type Ca^2+^ influx, inducing vasodilatation, elevated arterial compliance [50], and hypotension. For example, in the present study, levcromakalim, which causes hyperpolarization to V_K_ of −85 mV at 5.9 mM K^+^[47], caused a decline of resting intracellular Ca^2+^ and baseline tension, and shifted the [K^+^-contraction curve to higher K^+^ concentrations by +6 mM K^+^ at midpoint.

On the other hand, it is expected that factors causing depolarization of the membrane potential such as intravascular pressure [51], hypertension [2,52], a deficient NO release as in eNOS^−/−^ mice [45], the absence of TRPC6 channels [46] might force the VSMC V_m_ in the L-type Ca^2+^ channel window leading to increased window L-type Ca^2+^ influx, basal constriction, decreased arterial compliance, increased myogenic responses and hypertension.

Therefore, results of the present study indicate that the position of the L-type Ca^2+^ channel window along the voltage axis may have profound effects on basal and stimulated Ca^2+^ influx in VSMC, but also predict that shifts of the activation or inactivation curves of L-type Ca^2+^ channels affect vasoconstriction and/or dilatation. For example, BAY K8644, which shifts the L-type Ca^2+^ channel activation curve to hyperpolarized potentials [33,34], caused an increase of basal Ca^2+^ influx and tone (Figure 8, see also [30]). Furthermore, Bay K8644 shifted the [K^+^-response curve to lower K^+^ concentrations by about 8 mM at midpoint, independent of the presence of levcromakalim, indicating that both the position of the window on the voltage axis and the resting membrane potential determine the window contraction.

Finally, because a number of alternatively spliced isoforms of the calcium channel gene protein exist, the L-type Ca^2+^ channel population is not homogeneous. The isoforms display differences in tissue distribution, physiology, pharmacology and disease-related up- and/or down-regulation [14,41,42,53], but also show altered voltage-dependent activation and inactivation, thereby influencing window currents [54]. Hence, changes in the expression of the channel isoforms within the vascular tree [55] as can occur in hypertension [53] or atherosclerosis [14] may affect the position of the L-type Ca^2+^ channel window along the voltage axis with effects on basal and stimulated Ca^2+^ influx and blood vessel tone. Moreover, different splice variants can be expressed within a single blood vessel type and depending on the dominance of one or more isoforms, this may determine the electrophysiological properties of the Ca^2+^ channels [42,53,55,56].

### K^+^-induced Ca^2+^ sensitization

The “window” intracellular Ca^2+^ signal elicited by depolarization reached a steady-state at 200 s, whereas tension increased further at later time intervals. This pointed to a time-dependent and Ca^2+^-dependent Ca^2+^ sensitization, but after normalization of the contractile responses, there was no shift of the curves with time. Hence, the time-dependent Ca^2+^ sensitization was proportional to intracellular Ca^2+^, which is mainly determined by the extent of “steady-state” Ca^2+^ influx at each [K^+^. This is in line with recent data indicating that the depolarization-induced Ca^2+^ sensitization depends on Ca^2+^ entry [15,18-21] and with the results obtained with the Rho kinase inhibitors Y-27632 and HA 1077. Rho-kinase inhibition did not eliminate the bell-shape of the [K^+^-force curves, but emphasized its voltage-dependence. Therefore, both continuous Ca^2+^ influx and Ca^2+^-dependent Ca^2+^ sensitization are necessary to maintain contraction, whereby Ca^2+^ influx occurs independently from Ca^2+^ sensitization, but not vice versa.

### Limitations of the study

Voltage-clamp of multicellular aortic segments with electrophysiological techniques is impossible with current methods because of temporal and spatial voltage heterogeneity. Therefore, we clamped the aortic rings with extracellular K^+^ although the resting V_m_ is not solely determined by the K^+^ equilibrium potential (V_K_), especially at low K^+^[51] (see Additional file 1). Taking into account that levcromakalim hyperpolarized V_m_ of rat mesenteric arteries from −58 to −82 mV (hyperpolarization to V_K_) [47] and that in the present study levcromakalim shifted the [K^+^-force curve by +5.9 mM K^+^ at midpoint, V_m_ at normal K^+^ of 5.9 mM was calculated to be 19 mV less polarized than V_K_ (−66 mV instead of the Nernstian −85 mV); this is in good agreement with resting V_m_ of arterial SMCs mentioned in the literature [28,47] (see Additional file 1). At 20 and 50 mM K^+^, the difference between V_m_ and V_K_ further diminished from 19 to 7 and 3 mV. Therefore, clamping the segments with K^+^ was, in our hands, a good technique to restrain the resting V_m_ of the SMCs.

### Conclusions

Besides a phasic, fast transient Ca^2+^ and force component, depolarization of aortic segments of C57Bl6 mice with elevated extracellular K^+^ causes a tonic, slow Ca^2+^ and force component. Both components reflect the electrophysiological properties of L-type Ca^2+^ channels. The tonic force component could be attributed to window L-type Ca^2+^ influx, plays a prominent role in maintaining basal and stimulated intracellular Ca^2+^ and tension in mouse aorta, and together with Rho-kinase-mediated Ca^2+^ sensitizing may be of great importance for the (patho)physiology of conduit blood vessels. Hence, any modulation of L-type Ca^2+^ influx in VSMC is expected to affect endothelium-dependent and -independent Ca^2+^ mobilization and related vasomotor responses of blood vessels or arterial compliance. Window L-type Ca^2+^ influx may underlie the reduced relaxing efficacy of NO in mouse aorta when the contraction is elicited mainly via L-type Ca^2+^ influx [30]. Therefore, we conclude that every intervention (short or long term) that changes the resting V_m_ of the VSMC or the expression/properties of the population of L-type Ca^2+^ channels, favoring one or another isoform, might have implications for the window Ca^2+^ current, influx and contraction, for the sensitivity to L-type Ca^2+^ channel blockers and NO, for the arterial compliance and for the effects of hypertension on the cardiovascular system.

## Methods

### Aortic segments

The studies were approved by the Ethical Committee of the University of Antwerp, and the investigations conform to the Guide for the Care and Use of Laboratory Animals published by the US National Institutes of Health (NIH Publication No. 85–23, revised 1996). C57Bl6 mice (n = 72, food and water ad libitum, 12/12 light–dark cycle) were used at the age of 4 to 7 months. Animals were euthanized under pentobarbital anesthesia (sodium pentobarbital, 75 mg kg^-1^, i.p.). The thoracic aorta was carefully removed, stripped of adherent tissue and dissected systematically. Starting at the diaphragm, the ascending thoracic aorta was cut in segments of 2 mm width (5 to 6 segments). Vessels were immersed in Krebs Ringer solution (KR 37°C, 95% O_2_/5% CO_2_, pH 7.4) with (in mM): NaCl 118, KCl 4.7, CaCl_2_ 2.5, KH_2_PO_4_ 1.2, MgSO_4_ 1.2, NaHCO_3_ 25, CaEDTA 0.025, and glucose 11.1. When Ca^2+^ was omitted from the KR, 1 mM EGTA was added (further named 0Ca) and, hence, to restore 2.5 mM free Ca^2+^, 3.5 mM Ca^2+^ was added to 0Ca (further named 0–3.5Ca) from a 1.75 M CaCl_2_ stock. High K^+^- solutions were prepared by replacing NaCl with equimolar KCl.

To measure resting membrane potentials (V_m_) inverted (inside out) endothelium-denuded segments were mounted in the wire myograph, incubated with HEPES-buffered bathing solution (5.4 mM KCl, 141 mM NaCl, 10 mM HEPES, 0.8 mM MgCl_2_, 10 mM glucose, 1.8 mM CaCl_2_, 1 μM amlodipine, pH = 7.4 at 37°C with 1 M NaOH) and impaled with glass intracellular microelectrodes (filled with 2 mM KCl and tip resistances between 65 and 90 MΩ). V_m_ was measured with a HEKA EPC9 amplifier (HEKA Electroniks, Germany) in the zero current clamp mode and recorded on paper (Gould pen writer). Only measurements of V_m_ starting with a sharp decrease of V_m_ upon impalement and a sharp return to approximately 0 mV upon withdrawal of the electrode were considered.

To simulate voltage clamp protocols used in electrophysiological studies, extracellular K^+^ was used to clamp the aortic segments at certain estimated potentials. Depolarizing voltage steps were mimicked by graded elevation of extracellular K^+^ starting from and returning to a normal resting potential at 5.9 mM K^+^ (repetitive depolarization protocol). The holding potential from which voltage steps would be applied was mimicked by holding the segments at each K^+^ concentration before a subsequent challenge with higher K^+^ (cumulative depolarization protocol).

### Isometric tension measurements

Aortic segments were mounted in 10 ml organ baths, tension (mN) was measured isometrically with a Statham UC2 force transducer (Gould) connected to a data acquisition system (Powerlab 8/30, ADInstruments, Spechbach, Germany) as described [30]. Segments were gradually stretched until a stable loading tension of 16 mN, the optimal preload to attain maximal force development by 50 or 124 mM K^+^. Isometric force was reported in mN. Nitric oxide (NO) formation was inhibited with a combination of 300 μM *N*^Ω^-nitro-L-arginine methyl ester (L-NAME) and 300 μM *N*^Ω^-nitro-L-arginine (L-NNA) and to avoid any vasomotor interference due to prostanoids, 10 μM indomethacin was present.

### Combined assay of isometric tension and VSMC Ca_i_^2+^

Segments were mounted in a wire (40 μm) myograph above an inverted microscope (Axiovert 200, Carl Zeiss, Zaventem, Belgium) after removal of the endothelium by rubbing their interior with a braided silk wax to avoid interference by endothelial Ca^2+^ signals. Segments were loaded for 120 minutes with aerated (95% O_2_/5% CO_2_, pH 7.4) KR containing 10 μM Fura-2 AM, 1 mg/ml bovine serum albumin and 0.02% Pluronic at room temperature. Then, temperature was raised to 37°C and the segment was set to its normalized diameter [30]. The single emission (510 nm) ratio at dual excitation (340 and 380 nm) was used as a relative measure of free Ca_i_^2+^ (relative units, RU) after subtraction of background emission values, which were determined by adding 2 mM MnCl_2_ at the end of each experiment. Contractile force was measured simultaneously and reported in mN mm^-1^[30].

### Data analysis

All results are expressed as mean ± sem; n represents the number of mice. Time-force curves were fitted with a bi-exponential function revealing amplitudes and time constants of first (fast) and second (slow) components. Concentration-response curves were fitted with sigmoidal concentration-response equations with variable slope, which revealed maximal responses (E_max_) and the negative logarithm of the concentration resulting in 50% of the maximal effect (pEC_50_) for each vessel segment. Two-way ANOVA with Bonferroni post-test (concentration-response curves) and paired or unpaired *t*-test (GraphPad Prism, version 5, GraphPad Software, San Diego California USA) were used to compare means of the different experimental groups. A 5% level of significance was selected.

### Materials

Sodium pentobarbital (Nembutal®) was obtained from Sanofi (Brussels, Belgium), indomethacin from CERTA (Belgium), L-NNA, L-NAME, nifedipine, ryanodine, 2-APB, HA-1077 dihydrochloride from Sigma (Bornem, Belgium), Fura 2-AM from Molecular Probes (Invitrogen, Merelbeke, Belgium), (±) BAY K8644, levcromakalim, glibenclamide from TOCRIS (Bristol, United Kingdom), Y-27632 dihydrochloride from Abcam Biochemicals (Cambridge, UK).

## Abbreviations

Ca^2+^: Calcium; VSMC: Vascular smooth muscle cell; K^+^: Potassium; 2-APB: 2-aminoethoxydiphenyl borate; L-NAME: *N*^Ω^-nitro-L-arginine methyl ester; L-NNA: *N*^Ω^-nitro-L-arginine; NO: Nitric oxide; SERCA: Sarco-endoplasmic reticulum calcium ATPase; SR: Sarcoplasmic reticulum; eNOS: Endothelial nitric oxide synthase; V_K_: Equilibrium potential for K^+^ ions; V_m_: Membrane potential.

## Competing interests

The authors declare that they have no competing interests.

## Authors’ contributions

All authors read and approved the final manuscript. PF and CVH conceived of the study, designed the experiments, collected and analyzed the data; PF and HB drafted the manuscript, PF, CVH, JVL, DS, WM, GDM and HB participated in interpretation of the results and final draft of the manuscript.

## Supplementary Material

Additional file 1 Additional information. Click here for file

## Author details

^1^Laboratory of Physiopharmacology, University of Antwerp, Universiteitsplein 1 Building T, 2.18, Wilrijk B-2610, Belgium. ^2^Laboratories of Pharmacology, University of Antwerp, Antwerp, Belgium.
