# Supplementary material for: Contribution of transient and sustained calcium influx, and sensitization to depolarization-induced contractions of the intact mouse aorta
Source: BMC Physiol. 2012 Sep 3;12:9. doi: 10.1186/1472-6793-12-9 (PMC3499395; doi:10.1186/1472-6793-12-9)
Supplement: Additional file 1 — Additional information. [file 1472-6793-12-9-S1.docx]

**Additional information**

**Contribution of transient and sustained calcium influx, and Ca^2+^-sensitization to depolarization-induced contractions of intact mouse aorta.**

**1. Resting membrane potentials (V_m_) of vascular smooth muscle cells (VSMCs) in aortic segments.**

According to Knot and Nelson [1], the relationship between extracellular K^+^ and V_m_ of VSMCs is Nernstian above 16 mM [K^+^]. Figure 8 of the manuscript reveals that 200 nM levcromakalim shifted the K^+^ dose-response (KDR) at half maximal effective K^+^ concentration in control by 5.93±0.87 mM K^+^ to higher extracellular K^+^ concentrations. This shift will probably underestimate the real shift because the effects of levcromakalim are expected to be larger at lower extracellular K^+^ (at 25% of the maximum this shift was 6.58±0.68 mM K^+^). Nevertheless, if one takes into account the “underestimated” shift of +5.93 mM K^+^ of the KDR than the relationship between V_m_ and the extracellular K^+^ can be estimated. Taking into account the Nernstian V_K_ of 61*log([K^+^]_o_/[K^+^]_I_, V_m_ can be estimated as 61*log(([K^+^]_o_ + 5.93)/[K^+^]_i_)) with [K^+^]_o_ the extracellular K^+^ concentration in mM and [K^+^]_i_ the intracellular K^+^ concentration (assumed to be 145 mM). Figure 1 displays the relationship between estimated and measured V_m_. The slope of this relationship was not significantly different from unity indicating that estimated V_m_ agreed very well with measured V_m_. According to the estimation, V_m_ at 5.9 mM K^+^ is around -65 mV instead of the Nernstian -85 mV, which is in good agreement with measured V_m_ of -60.1±2.6 mV (8 measurements in 3 mice, figure 1). 200 nM Levcromakalim hyperpolarised V_m_ from -64 to -86 mV. Values of V_m_ measured in mouse aortic segments agreed well with similar measurements in VSMCs from different arteries (-53.5±3.5 mV, bovine choroidal artery, n=4 [2]; -59±3 mV, mouse pulmonary artery, n=5 [3]; -40 to -50 mV, rat mesenteric arteries, [4]; -54.7±0.3 mV, rat pulmonary artery, n=13 [5] and -51.1±1.3 mV, guinea pig internal carotid artery, n=19 [6]).


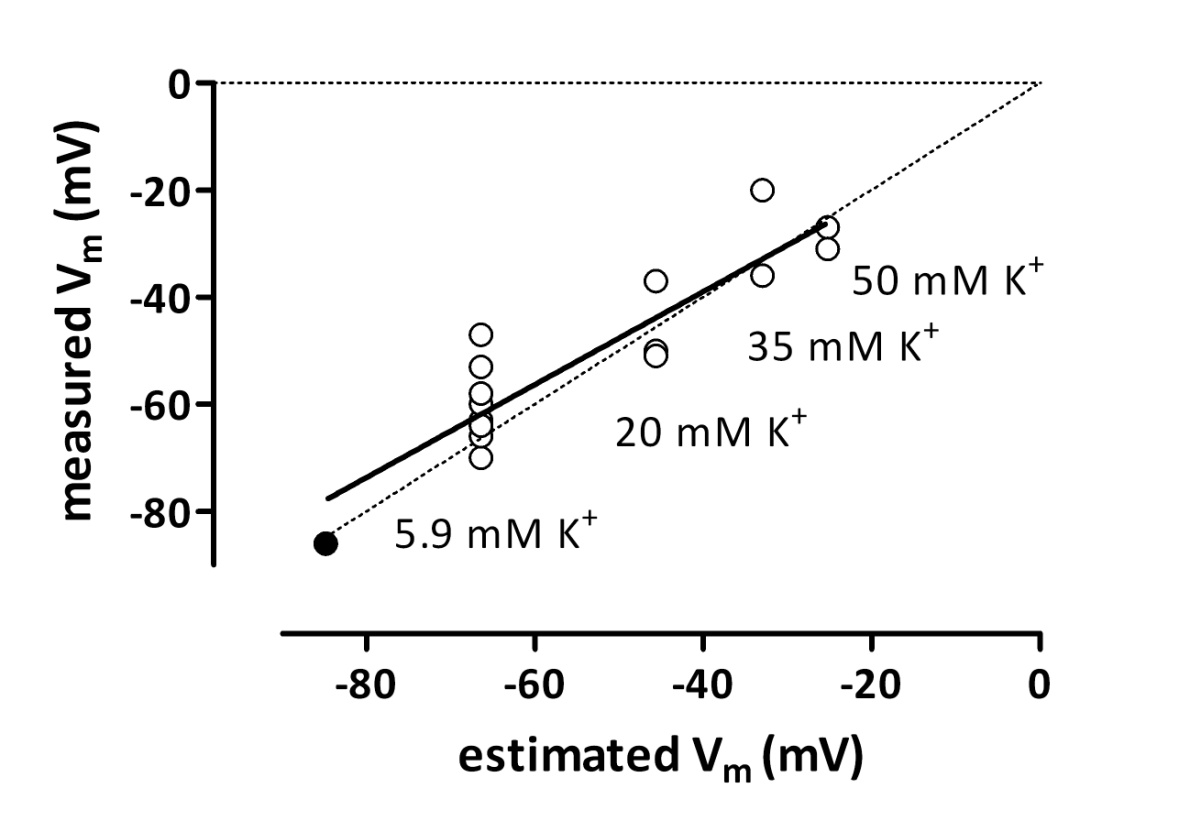


**Figure 1: Relationship between estimated and measured membrane potential (V_m_) of mouse aortic VSMCs.** Linear regression of the relationship between estimated and measured V_m_ (open circles, straight line) reveals slope of 0.90 (95% confidence interval 0.69-1.12), which was not significantly different from unity (dotted line) (R^2^= 0.84). Full circle represents V_m_ in the presence of 200 nM levcromakalim at 5.9 mM K^+^.

**2. Absence of neurogenic contribution to contraction**

To exclude the possible contribution of neurotransmitter release from perivascular nerves to high K^+^-contractions, the following experiments were performed. In arterial segments of 3 mice we applied 300 ng/ml 6-hydroxy-dopamine (6-HODA) to release stored neurotransmitter [7]. 6-HODA caused no contraction of aortic segments, but contracted femoral artery segments of the same mice (positive control, figure 2A). Similarly, 1µM capsaicin, which is known to activate TRPV1 channels in afferent nerves [8] caused transient contractions of the femoral artery, but not of aortic segments (figure 2B). Third, electrical field stimulation (40V, 2 – 20 Hz) caused frequency-dependent (figure 2C), and TTX- and prazosin (both 1 µM)-sensitive force development in the femoral artery, but not in aortic segments of the same mice. Finally, the K^+^-dose-response relationships in mouse aortic segments in control, after emptying presynaptic neuronal vesicles with 300 ng/ml 6-OH-dopamine, after inhibition of nerve action potentials with 1 µM TTX or after inhibition of α-adrenoceptors with 1 µM prazosin were very similar (figure 2D).

**
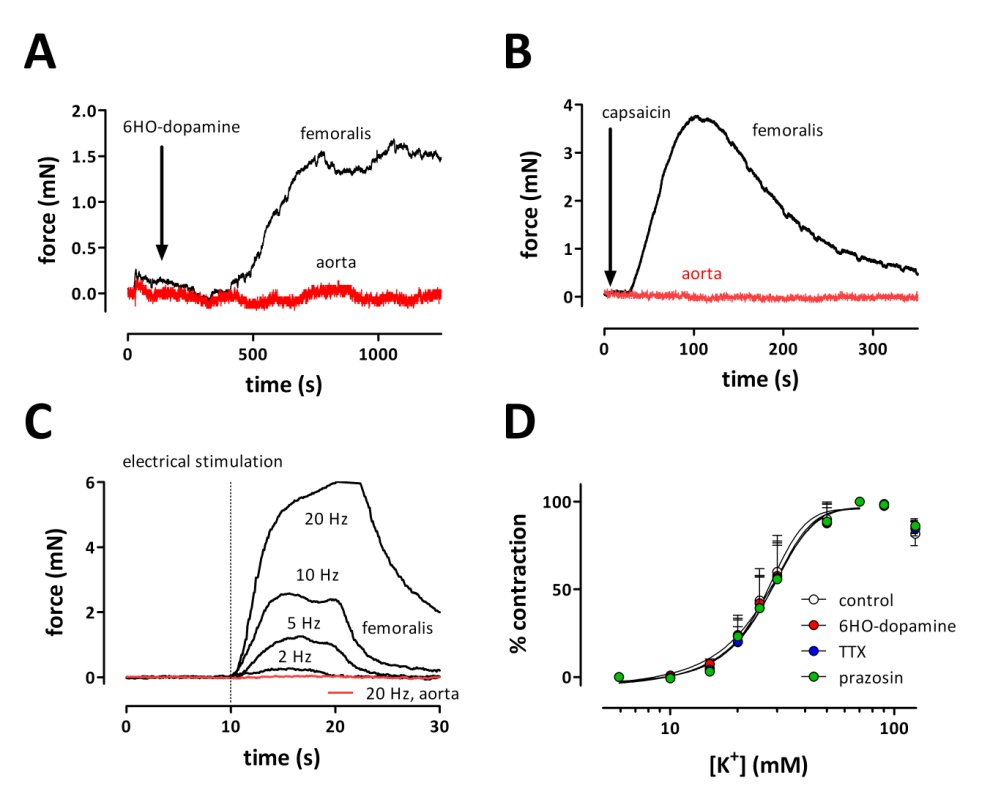
**

**Figure 2: Absence of neurogenic influence on K^+^ contractions in aorta.** Effects of 6HO-dopamine (A) and 1 µM capsaicin (B) on basal tension in arteria femoralis (black) and aorta (red) of the same mouse (representative examples of 3 experiments). C: Electrical field stimulation (2-20 Hz) elicits frequency-dependent forces in arteria femoralis (black), but not in aorta (red, 20 Hz shown). D: Effects of 300 ng/ml 6HO-dopamine, 1 µM TTX and 1 µM prazosin on K^+^ dose-response curves (relative values with force at 70 mM K^+^ as 100%) in aortic segments of 4 mice (mean±SEM).

These experiments indicate that there is no significant contribution of neurotransmitter release from perivascular nerves to depolarization(K^+^)-induced responses of mouse aortic segments.

Reference List

1. Knot HJ, Nelson MT: **Regulation of arterial diameter and wall [Ca^2+^] in cerebral arteries of rat by membrane potential and intravascular pressure.** *J Physiol* 1998, **508 ( Pt 1):**199-209.

2. Delaey C, Boussery K, Breyne J, Vanheel B, Van d, V: **The endothelium-derived hyperpolarising factor (EDHF) in isolated bovine choroidal arteries.** *Exp Eye Res* 2007, **84:**1067-1073.

3. Tolsa JF, Marino M, Peyter AC, Beny JL: **Role of membrane potential in endothelium-dependent relaxation of isolated mouse main pulmonary artery.** *J Cardiovasc Pharmacol* 2006, **47:**501-507.

4. Kwan HY, Shen B, Ma X, Kwok YC, Huang Y, Man YB, Yu S, Yao X: **TRPC1 associates with BK(Ca) channel to form a signal complex in vascular smooth muscle cells.** *Circ Res* 2009, **104:**670-678.

5. Gonczi M, Szentandrassy N, Johnson IT, Heagerty AM, Weston AH: **Investigation of the role of TASK-2 channels in rat pulmonary arteries; pharmacological and functional studies following RNA interference procedures.** *Br J Pharmacol* 2006, **147:**496-505.

6. Gluais P, Edwards G, Weston AH, Vanhoutte PM, Feletou M: **Hydrogen peroxide and endothelium-dependent hyperpolarization in the guinea-pig carotid artery.** *Eur J Pharmacol* 2005, **513:**219-224.

7. Azuma H, Ishikawa M, Nakajima T, Satoh A, Sekizaki S: **Calcium-dependent contractile response of arterial smooth muscle to a jellyfish toxin (pCrTX: Carybdea rastonii).** *Br J Pharmacol* 1986, **88:**549-559.

8. Martinez AC, Pagan RM, Prieto D, Recio P, Garcia-Sacristan A, Hernandez M, Benedito S: **Modulation of noradrenergic neurotransmission in isolated rat radial artery.** *J Pharmacol Sci* 2009, **111:**299-311.
